# The Effect of Oral Citicoline and Docosahexaenoic Acid on the Visual Field of Patients with Glaucoma: A Randomized Trial

**DOI:** 10.3390/life12101481

**Published:** 2022-09-23

**Authors:** Alfonso Anton, Virginia Garcia, Marcos Muñoz, Karla Gonzales, Eleonora Ayala, Estela del Mar Sanchez, Antonio Morilla-Grasa

**Affiliations:** 1 Institut Catala de Retina, Universitat Internacional de Catalunya, 08017 Barcelona, Spain; 2 Institut Catala de Retina, 08022 Barcelona, Spain

**Keywords:** glaucoma, treatment, nutraceuticals, docosahexaenoic acid, citicoline, vitamin C

## Abstract

The role of nutraceuticals in the treatment of glaucoma remains controversial. The aim of this study was to evaluate the effect of citicoline, vitamin C, and docosahexaenoic acid (DHA) in patients with glaucoma. Methods: This was a prospective, randomized study. Patients with glaucoma were randomized to one of four groups and treated for 3 months with vitamin C, DHA, citicoline, or a combination of DHA and citicoline. We conducted a complete ophthalmic examination and visual fields each month and calculated the slopes of field indices. Changes in visual field indices (VFIs) and their slopes were assessed in each group and compared. Results: Seventy-three persons were included in the study. Mean defect (MD) significantly improved (*p* = 0.001) from −9.52 ± 4.36 to −7.85 ± 4.36 dB during the study period in persons taking DHA + citicoline. Similarly, the mean VFI significantly improved (*p* = 0.001) in this group. The only treatment group showing a statistically significant improvement (*p =* 0.006) in the MD (from −0.1041 ± 0.2471 to 0.1383 ± 0.2544 dB/month) and VFI slope was the group treated with DHA+citicoline. Conclusions: The combination of oral treatment with DHA + citicoline significantly improved VF indices and their slopes in patients with glaucoma after 3 months of treatment.

## 1. Introduction

Glaucoma is a progressive optic neuropathy that affects 3.54% of the population aged 40–80 years, and it is estimated that it will affect over 111 million people by 2040 [1]. In addition, glaucoma is the second leading cause of blindness, accounting for 14% of blindness causes [2]. Current scientific evidence indicates that lowering intraocular pressure (IOP) with medical, laser, and/or surgical treatment is effective in decreasing the risk of disease progression [3,4,5,6,7]. Nevertheless, several randomized clinical trials and clinical experience have shown that despite significant IOP lowering, some patients continue to show worsening optic nerve damage. For example, 45% of early glaucoma cases progressed in the treatment group of the Early Manifest Glaucoma Treatment Trial [4], and 20% of normal tension glaucoma worsened despite medical treatment in the Collaborative Normal Tension Glaucoma Study [7]. This clinical evidence supports the multifactorial physiopathology that is widely accepted for glaucoma. 

During the last few decades, considerable effort has been made to identify useful medical treatments for glaucoma that could complement IOP-lowering therapies. The recently released European Glaucoma Guidelines [8] state that there is no scientific evidence to support neuroprotective agents in the treatment of glaucoma. In contrast to this assertion, many patients with glaucoma are recommended to take nutraceuticals containing vitamins, citicoline, or ginkgo biloba, among others, in clinical practice. There are probably several reasons for the lack of evidence, including the difficulty of demonstrating neuroprotection in clinical studies, a lack of interest among pharmaceutical companies in performing complex and expensive studies on substances not protected by a patent, or, simply, an effective neuroprotective or neuromodulator agent has not yet been identified. Regardless of the reasons for the lack of evidence, the role of treatments other than IOP lowering remains to be determined, and there is a need for well-designed randomized controlled trials. 

Citicoline is an indispensable intermediary in the synthesis of cell membrane phospholipids and a potential neuroprotectant or neuromodulator used in some countries to facilitate recovery from stroke lesions or for the treatment of neurodegenerative diseases [9]. The substance is well-tolerated, and there is limited evidence in small studies suggesting a possible positive effect on visual function in patients with glaucoma [9,10,11]. Docosahexaenoic acid (DHA) is an omega-3 polyunsaturated fatty acid with demonstrated antioxidant properties [12], with limited evidence on its potential beneficial effects in patients with glaucoma [12,13].

The present study aimed to evaluate the effect of citicoline and DHA on the visual field (VF) of patients with glaucoma.

## 2. Materials and Methods

### 2.1. Design

This as a prospective, randomized, single-blind study. 

### 2.2. Ethics

The study was approved by the Research Commission of our institution and the Ethics Committee. All participants signed an informed consent form after being informed of the nature of the study. 

### 2.3. Sample

Seventy-three participants with chronic glaucoma, aged 50 to 75 years, were included in the study and randomly assigned to one of four treatment groups (see below). All participants had glaucomatous optic nerve damage and glaucomatous VF defect. Glaucomatous optic neuropathy was defined as the presence of optic disc rim thinning, peripapillary hemorrhage, abnormally thin retinal nerve fiber layer (RNFL) as measured with optical coherence tomography (OCT), and/or the concomitant presence of glaucomatous VF defect. A glaucomatous VF defect was considered if a minimum of three contiguous locations outside the 95% normal limits in the pattern deviation plot on two consecutive fields was detected. Participants were included in the study if they could perform reliable VFs with false-positive responses below 20% and false-negative responses below 30%.

Participants were excluded from the study if they were taking any other nutraceuticals, had any other condition that could alter the VF, had hypersensitivity to aspirin or fish oil, had undergone intraocular surgery in the 6 months before entering the study, or required significant changes in hypotensive treatment (addition of a drug of a different type, any laser treatment or any surgical treatment) during the course of the study. Only one eye per person was included in the study. If both eyes met the selection criteria, the worst eye, based on MD value, was selected.

### 2.4. Examinations

At baseline, participants underwent a complete ophthalmic examination including refraction, anterior and posterior pole evaluation, gonioscopy, two VFs in a maximum period of 6 months, two OCT peripapillary images, pachymetry and two IOP measurements with Goldman applanation tonometry. The VF was examined with the 24-2 SITA standard test of a Humphrey Field Analyzer (Zeiss-Meditec, Dublin, CA, USA), and OCT was examined with the optic disc protocol of Cirrus OCT (Zeiss-Meditec, Dublin, CA, USA). All participants were examined again at 1, 2, and 3 months. Again, a complete ophthalmic examination was performed, including VFs, at each visit and OCT images at the final visit at 3 months.

### 2.5. Treatment Groups

Participants were randomly allocated to one of four treatment groups using the Excel (Microsoft, Seattle, WA, USA) randomization function. All participants took the assigned treatment for 3 months. Adherence was checked at each visit by the study coordinators, who assessed the blisters, boxes, and bottles of treatment used by the participants. The physicians and optometrists examining the patients were always blind to the treatment taken by the patient. The four treatment groups and dose regimen were as follows:

Group 1: Vitamin C, one 500 mg tablet per day (Solgar, Las Rozas, Madrid, Spain).

Group 2: DHA, three tablets per day (Brudypio 1.5 g, Brudylab SLU, Madrid, Spain). This tablet also included much lower amounts of eicosapentaenoic acid; docosapentaenoic acid; vitamins A, C, E, and B; minerals; and other components.

Group 3: Citicoline, two sachets per day (Cebrolux 800, Bausch&Lomb, Rochester, NY, USA).

Group 4: DHA, three tablets per day (see above) plus citicoline, two 800 mg sachets per day (see above).

### 2.6. Statistical Analysis

The Shapiro–Wilk test was used to assess the normal distribution of the variables. Mean defect (MD) and visual field index (VFI) slopes were calculated before treatment initiation (pretreatment slope) and after 3 months of treatment (post-treatment slope). Post-treatment slopes were assessed using two baseline VFs and the three follow-up fields. Additionally, whenever possible, a pretreatment slope of field indices was calculated using the last five fields performed by the patient before initiating study treatment. This subgroup of 66 participants allowed comparison between pre- and post-treatment slopes. The *t*-test (paired) or Wilcoxon test was used to compare pre- and post-treatment VF indices and slopes within each group. The Kruskal–Wallis test was used to compare pre- and post-treatment MD and VFI slopes among the four different groups. The change in MD and VFI slope after treatment initiation was calculated by subtracting the pretreatment slope from the post-treatment slope. Slopes were considered to have improved with treatment if the result of the subtraction was positive and worsened with treatment if the result was negative. The percentage of patients with slope improvement during study time was compared among the groups. For all analyses, a *p*-value under 0.05 was considered significant.

## 3. Results

### 3.1. Sample

**Figure 1:**
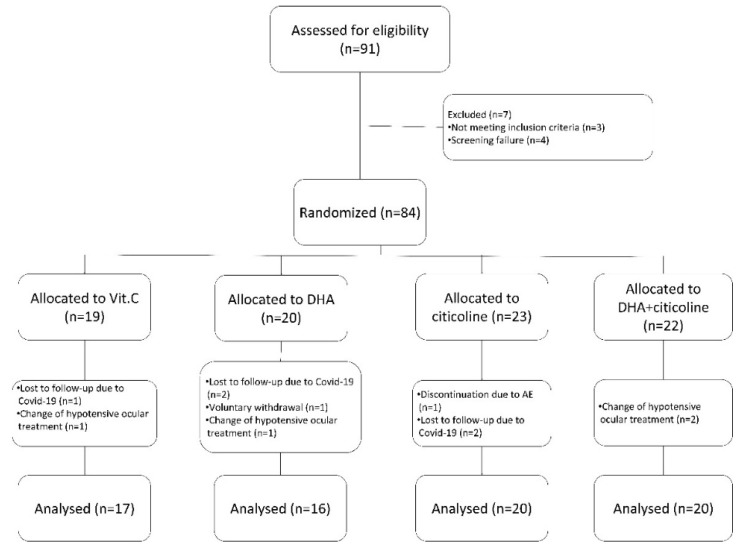
Patient selection diagram. Of the initially prescreened 91 patients, 73 were finally included in the study and underwent randomization and treatment.

The sample selection process is shown in Figure 1. A total of 73 participants were included in the study: 17 in group 1 (vitamin C), 16 in group 2 (DHA), 20 in group 3 (citicoline), and 20 in group 4 (DHA+citicoline). The sample characteristics are shown in Table 1. To ensure homogeneous age throughout the groups, the patients were stratified into two age groups (50–64 and 65–75 years). There were no significant differences among the different treatment groups with regard to age; sex; type of glaucoma; or baseline MD, VFI, or OCT RNFL. Most (n = 61 (83.6%)) participants had primary open-angle glaucoma (POAG).

### 3.2. Change in IOP, MD, and VFI Slopes with Treatment

Mean IOP did not change significantly from baseline (13.96 ± 3.1 mmHg) to month 3 (14.03 ± 3.1 mmHg), globally or in any of the study groups. There were no statistically significant differences in baseline IOP or IOP at month 3 among the groups. Baseline and 3-month VF indices are detailed in Table 2. Mean MD significantly improved (*p* = 0.001) from −9.52 ± 4.36 to −7.85 ± 4.36 dB during the study period in group 4 (DHA + citicoline). Similarly, mean VFI also significantly improved (*p* = 0.001) from −75.15 ± 13.76% to 78.90 ± 14.82% with treatment in group 4. There were no significant differences in the VFI during the study in any other treatment group, but MD significantly improved, and VFI showed a nonsignificant tendency to increase in the overall sample.

### 3.3. Visual Field Slopes during Treatment Period

Visual field MD and VFI slopes are shown in Table 3. No statistically significant differences (Kruskal–Wallis Test) among the four groups were found during the pretreatment period in MD slopes (*p* = 0.702) or VFI slopes (*p* = 0.555). All treatment groups showed a mean positive slope, for MD and VFI, during the 3 months of treatment. Posttreatment MD slopes in the groups ranged from 0.02 dB/month (estimated 0.24 dB/year) to 0.13 dB/month (estimated 1.56 dB/year). Post-treatment VFI slopes ranged from 0.01%/month (estimated 0.12%/year) to 0.27%/month (estimated 3.2%/year). 

### 3.4. Comparison between Pre- and Post-Treatment Slopes in Each Group

**Figure 2:**
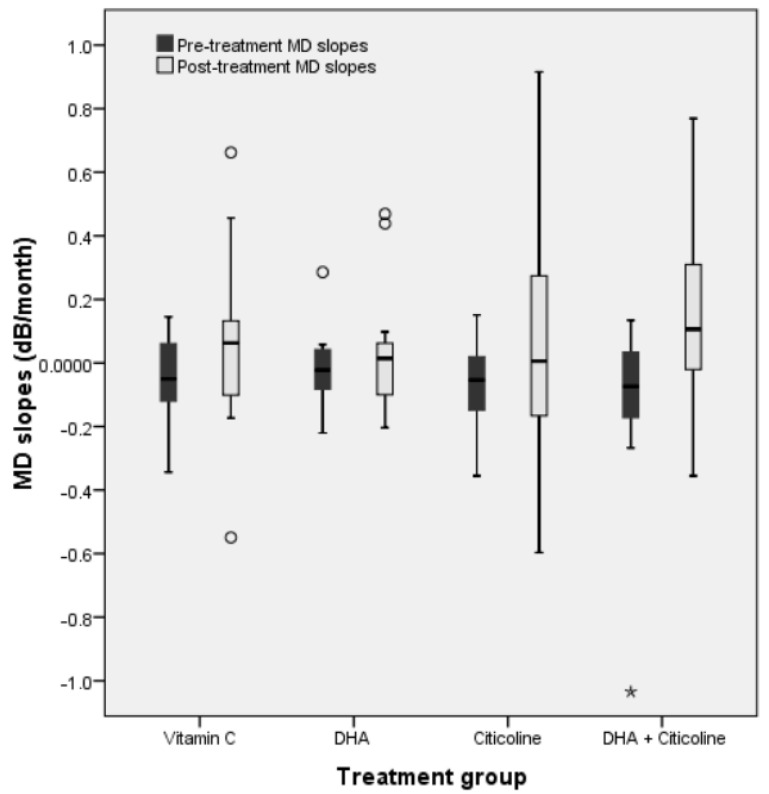
Comparison between mean MD slopes before the study and after treatment initiation in all four treatment groups. The only group that showed a statistically significant improvement (asterisk, *p* = 0.006) in D slopes was group 4 (DHA + citicoline).

**Figure 3:**
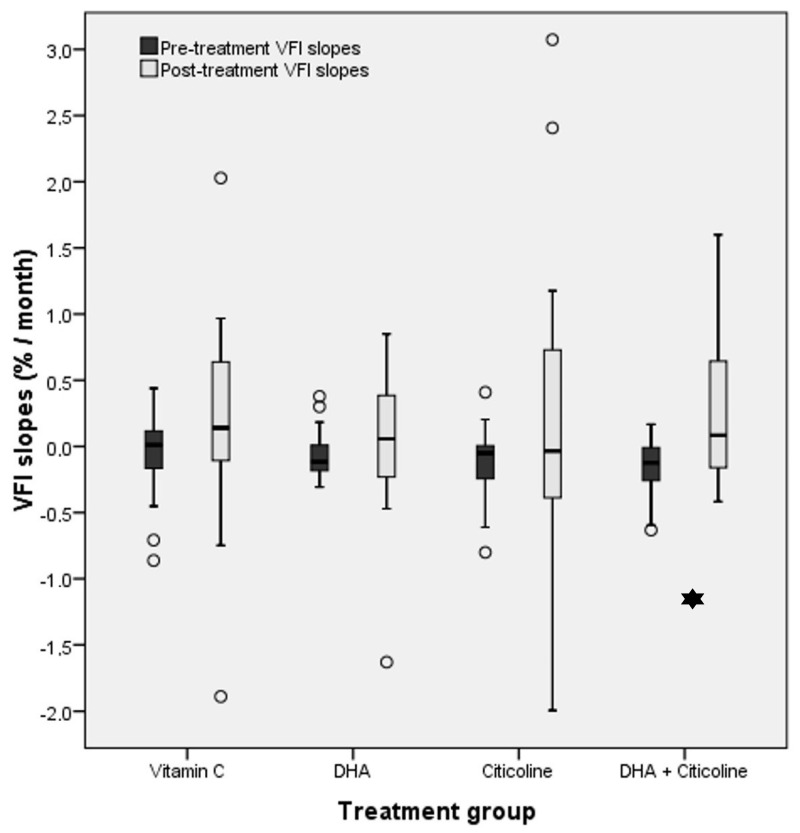
Comparison between mean VFI slopes before the study and after treatment initiation among the four treatment groups. Comparison between mean VFI slopes before the study and after treatment initiation in all four treatment groups. The only group showing a statistically significant improvement (asterisk, *p* = 0.006) in VFI slopes was group 4 (DHA + citicoline).

Changes in VFI slopes with treatment are shown in Table 3 and Table 4 and Figure 2 and Figure 3. All groups and indices showed a slightly negative slope during the pretreatment period and a positive slope during treatment (see the discussion for possible explanations). The only treatment group showing a statistically significant improvement (*p* = 0.006) in the MD and VFI slopes was group 4 (DHA + citicoline). The mean MD slope improved from −0.1041 ± 0.2471 to 0.1383 ± 0.2544 dB/month. The mean VFI slope improved from −0.1557 ± 0.2310%/month to 0.2780 ± 0.5661%/month in group 4. In both VF indices, the improvement could also be considered clinically significant because an approximate estimation of post-treatment yearly slopes would be 1.56 dB/year for MD and 3.24%/year for VFI. 

The positive or negative change in slope was also assessed by subtracting the post-treatment slope from the pretreatment slope (Table 4). Overall, 40 (MD) and 39 (VFI) persons had a positive result (improvement of the slope), and 26 (MD) and 27 (VFI) had a negative result (worsening of the slope), but the difference between positive and negative changes was not statistically significant. There were a significantly higher number of persons with MD slope improvement (16 vs.4, *p* = 0.007) and a nonsignificant tendency for a greater number of persons with VFI slope improvement (14 vs. 6, *p* = 0.076) in group 4 (DHA + citicoline).

## 4. Discussion

Many compounds, minerals, vitamins, and other substances with different potential actions have been tested as additional treatments for glaucoma, complementary to IOP lowering. The available evaluation of nutritional supplements for glaucoma is far from ideal because most studies had a short follow up and a small sample; many lacked a control group; and most did not measure the bioavailability of the compound in serum or in the eye. Despite the limited solid evidence, over 10% of patients with glaucoma were already taking some type of nutritional supplement in 2012 [14]. Among those taking supplementary nutrients, 34% took herbal medications; 22% applied dietary modifications; and 18% took combinations of vitamins, minerals, and other substances. 

Citicoline has been tested intramuscularly [15], orally [16], and in eyedrops [10,11] as a supplementary treatment for glaucoma with diverse results. DHA has been shown to decrease IOP in normal eyes [17], in patients with glaucoma [18,19], and to have an antioxidant effect in plasma [18,20]. To the best of our knowledge, this is the first prospective, randomized study comparing the effect of citicoline, DHA, and the association of both on the visual function of patients with glaucoma. 

Probably due to the methodical limitations mentioned above, the results of studies assessing nutritional supplements for the treatment of glaucoma are inconsistent, and researchers frequently did not use VFs to evaluate their effect. The latter would be desirable because this is the best, although imperfect, clinical estimate of the functional state of the optic nerve. In addition, it is probable that most studies with negative results have not been published, thus introducing an important bias in the limited evidence on nutritional supplements and glaucoma. 

A combination of homotaurine; forskolin root extract; l-carnosine; folic acid; vitamins B_1_, B_2,_ and B_6_; and magnesium for 12 months improved pattern electroretinogram amplitude and foveal sensitivity in patients with glaucoma compared with a control group [21]. Oral nicotinamide, a precursor NDA^+^ with a potential protective effect on retinal ganglion cells, has been found to improve photopic negative response [22] and VFs [23] in patients with glaucoma. Oral nicotinamide also showed improvement in a greater number of test locations than the placebo and a trend toward a better rate of progression of pattern standard deviation. 

Garcia-Medina et al. [24] compared two different antioxidant nutritional supplements including vitamins A, B, C, and E; and minerals, where one supplement contained a minimum dosage of DHA (96 mg), and found no significant change in the VF of patients with glaucoma. Ren et al. [25] observed a decrease in DHA levels in patients with glaucoma, while Yu et al. [26] reported greater functional damage in normal tension glaucoma with lower levels of serum DHA. In contrast, Yuki et al. [27] reported a lack of relationship between low levels of serum omega-3 fatty acids and normal tension glaucoma. 

Oral citicoline was shown to decrease the progression rate of MD of VF from −1.1 to −0.15 dB/year after 2 years of treatment [16] with a very modest simultaneous IOP reduction. The latter could be explained by the better compliance of patients participating in a study or by changes in IO-lowering treatment during the study. Eyedrops containing citicoline increased the amplitude of pattern electroretinogram and shortened the P100 implicit times of visual evoked potentials [10]. Parisi et al. [10] showed that this effect is reversible and stops after interrupting citicoline intake. More recently, Rossetti et al. [11] reported a lower rate of progression of MD of VF in patients with citicoline eyedrops (−1 dB/year) than in the placebo group (−1.9 dB/year), together with a lower loss of RNFL thickness in the treatment group (1.8 vs. 2.9 microns). 

Our results do not confirm the significant improvement in VF indices or the rate of progression in the citicoline group. The reasons for the inconsistent results for citicoline alone may lie in differences in study designs, different administration routes, or the bioavailability of the drug in each treatment regimen. Routine clinical experience suggests that some patients with glaucoma feel more able to perform certain activities, as well as VFs, when they take some nutritional supplements. A possible explanation is that the effect of these compounds may be heterogeneous among different persons due to unknown factors present in some and not in others. If this were the case, it may also explain why studies performed with small samples of a few dozen patients show inconsistent results. 

In the present study, DHA did not significantly improve the VF of patients with glaucoma. Nevertheless, we did find a rapid change in the VF of persons simultaneously taking DHA and citicoline, suggesting that these two components may have a synergistic effect on ganglion cell function. It is difficult to explain the mechanism of the synergistic antioxidant and/or neuro-modulatory effect of the association of citicoline and DHA. It could be speculated that both compounds boost each other in facilitating the rapid turnover of cell membrane structural components, contributing to maintaining its bilayer structure, which is needed for cellular communication and many other complex functions.

Our results do not confirm those reports showing a decrease in IOP values in patients taking DHA [18,19] or citicoline [11]. This is probably due to the fact that in the present study, contrary to other studies, patients who required significant changes in hypotensive treatment (addition of a drug of a different type, any laser treatment, or any surgical treatment) during the study were excluded. This was decided to prevent any bias in the assessment of the effect of DHA or citicoline on IOP or in the changes of VF indices or indices’ slopes, because it is well-known that a decrease in IOP significantly influences VF results, and it is likely to occur if hypotensive treatment is modified.

This study has limitations, such as a relatively small sample and a limited follow up. Progression rates in VF are better assessed in the longer term due to VF variability. Nevertheless, the sample was sufficiently large to identify significant changes in the VF during the treatment period and significant differences among the groups. Despite these limitations, the significant changes observed in group 4 (DHA + citicoline) suggest that this combination may have a positive synergistic effect on the visual function of patients with glaucoma. 

## 5. Conclusions

The combination of oral treatment with DHA + citicoline, in this relatively small sample, improved VF indices and their rate of progression in patients with glaucoma after 3 months of treatment. Longer studies with larger samples are desirable to confirm the significant VF improvement identified in this pilot study.

## Figures and Tables

**Table 1:** Sample characteristics.

	All Participants	Vitamin C	DHA	Citicoline	DHA +	*p* Value
					Citicoline	
Participants (n)	73	17	16	20	20	---
AGE						
(mean ± SD)	64.88 ± 6.20	65.94 ± 5.90	65.38 ± 5.93	64.80 ± 6.76	63.65 ± 6.33	0.716
−50–64: n (%)	31 (42.5)	7 (41.2)	7 (43.8)	8 (40.0)	9 (45.0)	1
−65–75: n (%)	42 (57.5)	10 (58.8)	9 (56.2)	12 (60.0)	11 (55.0)	
Sex:						0.391
Female: n (%)	34 (46.6)	9 (52.9)	8 (50.0)	6 (30.0)	11 (55.0)	
Mal: n (%)	39 (53.4)	8 (47.1)	8 (50.0))	14 (70.0)	9 (45.0)	
Right eye: n (%)	35 (47.3)	7 (41.2)	7 (43.8)	9 (45.0)	12 (60.0)	0.668
Type of glaucoma: n (%)						
- POAG						0.664
- PACG	61 (83.6)	14 (82.4)	14 (87.5)	18 (90.0)	15 (75.0)	
- NTG	2 (2.7)	0 (0.0)	1 (6.3)	0 (0.0)	1 (5.0)	
- Secondary	3 (4.1)	0 (0.0)	0 (0.0)	1 (5.0)	2 (10.0)	
	7 (9.6)	3 (17.6)	1 (6.2)	1 (5.0)	2 (10.0)	
Baseline IOP (mmHg) (mean ± SD)	13.96 ± 3.1	65.59 ± 9.72	67.28 ± 9.84	62.70 ± 12.69	65.05 ± 10.33	0.647
Baseline RNFL (microns) (mean ± SD)	65.01 ± 10.72	65.59 ± 9.72	67.28 ± 9.84	62.70 ± 12.69	65.05 ± 10.33	0.647
Baseline MD (dB)	−8.96 ± 3.91	−8.46 ± 3.70	−9.08 ± 4.07	−8.74 ± 3.70	−9.52 ± 4.36	0.863
(mean ± SD)						
Baseline VFI (%)	77.47 ± 12.83	78.35 ± 12.97	78.13 ± 12.83	78.50 ± 12.46	75.15 ± 13.76	0.831
(mean ± SD)						

DHA, docosahexaenoic acid; SD, standard deviation; IOP, intraocular pressure; POAG, primary open-angle glaucoma; PACG, primary angle closure glaucoma; NTG, normal tension glaucoma; RNFL, mean retinal nerve fiber layer; MD, mean defect of visual field; dB: decibels; VFI, visual field index.

**Table 2:** Visual field parameters at baseline and after 3 months of treatment.

	Baseline MD (dB)	Month 3 MD (dB)	*p* Value	Baseline VFI (%)	Month 3 VFI (%)	*p* Value
Global (mean ± SD)	−8.96 ± 3.91	−8.42 ± 4.29	**0.025**	77.47 ± 12.83	78.52 ± 13.94	0.096
Vitamin C (mean ± SD)	−8.46 ± 3.70	−8.32 ± 3.87	0.727	78.35 ± 12.97	78.53 ± 13.24	0.871
DHA (mean ± SD)	−9.08 ± 4.07	−8.86 ± 5.07	0.685	78.13 ± 12.83	77.56 ± 15.72	0.719
Citicoline (mean ± SD)	−8.74 ± 3.70	−8.71 ± 4.17	0.957	78.50 ± 12.46	78.90 ± 13.16	0.687
DHA + Citicoline (mean ± SD)	−9.52 ± 4.36	−7.85 ± 4.36	**0.001**	75.15 ± 13.76	78.90 ± 14.82	**0.008**

MD, mean defect of visual field; dB, decibels; VFI, visual field index; SD, standard deviation; DHA, docosahexaenoic acid.

**Table 3:** Comparison between pre- and post-treatment visual field index slopes in each group.

	MD Slopes (dB/Month)		*p* Value	VFI Slopes (%/Month)		*p* Value
	Pretreatment	Post-Treatment		Pretreatment	Post-Treatment	
Global (mean ± SD)	−0.0613 ± 0.1736	0.0867 ± 0.3092	**0.005**	−0.1107 ± 0.2781	0.1625 ± 0.8499	**0.018**
Vitamin C (mean ± SD)	−0.0502 ± 0.1459	0.055 ± 0.2895	0.350	−0.079 ± 0.3665	0.1425 ± 0.8992	0.485
DHA (mean ± SD)	−0.0135 ± 0.1116	0.0295 ± 0.1939	0.733	−0.0509 ± 0.1998	0.0150 ± 0.5971	0.532
Citicoline (mean ± SD)	−0.0624 ± 0.1307	0.1029 ± 0.4504	0.192	−0.1364 ± 0.3151	0.1733 ± 1.2439	0.371
DHA + Citicoline (mean ± SD)	−0.1041 ± 0.2471	0.1383 ± 0.2544	**0.006**	−0.1557 ± 0.2310	0.2780 ± 0.5661	**0.006**

MD, mean defect of visual field; dB, decibels; VFI, visual field index; SD, standard deviation; DHA, docosahexaenoic acid.

**Table 4:** Several patients with a positive or negative result in the subtraction of post-treatment slopes from pretreatment slopes per treatment group.

	Post-treatment—Pretreatment MD Slope			Post-treatment—Pretreatment VFI Slope		
	Positive (Number of Cases)	Negative (Number of Cases)	*p* Value	Positive (Number of Cases)	Negative (Number of Cases)	*p* Value
Global	40	26	0.085	39	27	0.140
Vitamin C	8	6	0.593	8	6	0.593
DHA	7	8	0.796	9	6	0.439
Citicoline	9	8	0.808	8	9	0.808
DHA + Citicoline	16	4	0.007	14	6	0.074

MD, mean defect of visual field; dB, decibels; VFI, visual field index; SD, standard deviation; DHA, docosahexaenoic acid.

## Data Availability

Not applicable.

## Registry

The study has been registered on www.clinicaltrials.gov. NCT05527106.
